# An ounce of prevention is worth a pound of cure: dreaded vascular complications during endonasal skull base surgery

**DOI:** 10.3171/2020.4.FocusVid.2019

**Published:** 2020-04-01

**Authors:** Aaron A. Cohen-Gadol

**Affiliations:** Associate Editor-in-Chief, *Neurosurgical Focus: Video*;1 Department of Neurosurgery, Indiana University, Indianapolis; and; 2 *The Neurosurgical Atlas*, Indianapolis, Indiana

Benjamin Franklin’s quote “An ounce of prevention is worth a pound of cure” resonates with all surgeons. Tumors displace cranial nerves and vessels, and injury to these vital structures is often most unexpected. Presumption is the opposite of prevention. It is okay to say, “There it is” and be wrong a hundred times, but it is not okay to say, “There it was” and be right even once.

In their video titled “Management of Arterial Injuries in Endoscopic Endonasal Approaches,” McDowell et al. demonstrate their virtuosity in managing two intraoperative vascular injuries. The first injury occurred at the level of the right ophthalmic artery while using Kerrison rongeurs. I am not convinced that exposure or bony removal along such a long proximal segment of the optic nerve is necessary; however, this injury was very well managed using bipolar electrocautery. The use of an air drill to eggshell the bone and fine curettes to mobilize the remaining bone would have been a reasonable strategy for minimizing this complication.

The second vascular injury, to the anterior cerebral artery, was more formidable and underscores the importance of adequate inspection while using microscissors. The adherent firm tumor can truly test the patience of a surgeon at the end of a long operative day. In addition, operative blind spots are expanded in endonasal surgery. In this case, the preoperative images show vascular encasement and lead one to consider a transcranial approach for handling this tumor; this approach might have facilitated safer vascular visualization/microdissection and potentially more complete tumor resection. Ultimately, the decision to leave a thin sheet of the tumor behind in this video was appropriate. Overall, the lessons from this case are significant and remind us that our failures are our most compelling teachers, providing us with essential perspectives.

